# Orexinergic innervations at GABAergic neurons of the lateral habenula mediates the anesthetic potency of sevoflurane

**DOI:** 10.1111/cns.14106

**Published:** 2023-02-05

**Authors:** Fang Zhou, Dan Wang, Huiming Li, Sa Wang, Xinxin Zhang, Ao Li, Tingting Tong, Haixing Zhong, Qianzi Yang, Hailong Dong

**Affiliations:** ^1^ Department of Anesthesiology and Perioperative Medicine, Xijing Hospital Fourth Military Medical University Xi'an China; ^2^ Anesthesia and Operation Center The First Medical Center of Chinese PLA General Hospital Beijing China; ^3^ Department of Anesthesiology, Ruijin Hospital Shanghai Jiao Tong University School of Medicine Shanghai China

**Keywords:** GAD2‐expressing neuron, general anesthesia, lateral habenula, orexin, sevoflurane

## Abstract

**Aims:**

The circuitry mechanism associated with anesthesia‐induced unconsciousness is still largely unknown. It has been reported that orexinergic neurons of the lateral hypothalamus (LHA) facilitate the emergence from anesthesia through their neuronal projections to the arousal‐promoting brain areas. However, the lateral habenula (LHb), as one of the orexin downstream targets, is known for its anesthesia‐promoting effect. Therefore, the current study aimed to explore whether and how the orexinergic projections from the LHA to the LHb have a regulatory effect on unconsciousness induced by general anesthesia.

**Methods:**

We applied optogenetic, chemogenetic, or pharmacological approaches to regulate the orexinergic^LHA‐LHb^ pathway. Fiber photometry was used to assess neuronal activity. Loss or recovery of the righting reflex was used to evaluate the induction or emergence time of general anesthesia. The burst‐suppression ratio and electroencephalography spectra were used to measure the anesthetic depth.

**Results:**

We found that activation of the orexinergic^LHA–LHb^ pathway promoted emergence and reduced anesthetic depth during sevoflurane anesthesia. Surprisingly, the arousal‐promoting effect of the orexinergic^LHA‐LHb^ pathway was mediated by excitation of glutamate decarboxylase (GAD2)‐expressing neurons, but not glutamatergic neurons in the LHb.

**Conclusion:**

The orexinergic^LHA–LHb^ pathway facilitates emergence from sevoflurane anesthesia, and this effect was mediated by OxR2 in GAD2‐expressing GABA neurons.

## Graphical abstract

During sevoflurane anesthesia, activation of the orexin innervations at GABA neurons of the LHb via OxR2 facilitates the emergence of mice; the emergence‐promoting effect of LHb GABA neurons may be mediated by their local effect in the LHb.

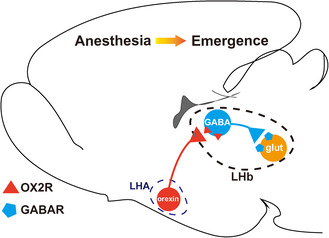

## 1 INTRODUCTION

General anesthesia has been used for more than 170 years^1^; however, the underlying mechanisms have not been fully elucidated.^2^, ^3^ Emerging evidence has revealed that neural circuits in the brain are involved in anesthesia‐induced unconsciousness.^4^, ^5^, ^6^ The lateral hypothalamus (LHA) has long been considered a conserved hub that integrates circadian rhythms, metabolic status, and emotion inputs to modulate arousal. Orexinergic neurons, exclusively located in the LHA, are involved in the implicated functions and play an essential role in promoting wakefulness. The orexinergic system conveys information to multiple output regions through the two peptides (orexin A [OA] and orexin B [OB]) and their widespread receptors (orexin receptor 1 [OxR1] and orexin receptor 2 [OxR2]). The extracellular levels of OAs have been reported to be high during the active wake state.^7^, ^8^, ^9^ Our lab and others using electroencephalogram (EEG) and behavioral testing have demonstrated that orexinergic neurons facilitate emergence from general anesthesia through their projections to the excitatory down‐stream neurons that promote emergence from anesthesia, such as the dopaminergic neurons of the ventral tegmental area and cholinergic neurons of the basal forebrain.^10^, ^11^

The lateral habenula (LHb) is one of the main downstream targets of orexinergic innervations.^12^, ^13^, ^14^ Notably, although the LHb is predominantly composed of the excitatory glutamatergic neurons, the LHb glutamatergic output is active during propofol‐ and isoflurane‐induced anesthesia, and activation of LHb glutamatergic neurons effectively shortens anesthesia induction and prolongs emergence from general anesthesia.^15^, ^16^ Therefore, whether the orexinergic neurons promote emergence via the anesthesia‐facilitating center of the LHb is not only an interesting question, but the underlying mechanisms would also supplement understanding for the networks between the arousal center and the anesthesia center. In this study, we used chemogenetic, optogenetic, EEG recordings, and behavioral tests to investigate the role of orexinergic^LHA–LHb^ projections in the regulation of consciousness and its neuronal mechanism in sevoflurane anesthesia, an extensively used volatile anesthetic.

## 2 METHOD

### 2.1 Animals

All experiments were in accordance with protocols approved by the Ethics Committee for Animal Experimentation and strictly abided by the Guidelines for Animal Experimentation of the Fourth Military Medical University. C57/BL6J male mice were obtained from Vital River Laboratory Animal Technology Co., Ltd. Glutamate decarboxylase 2 (GAD2)‐Cre male mice were purchased from Jackson Laboratory. Hcrt‐Cre male mice were the present from Luis de Lecea (Stanford University). Mice (aged 8–12 weeks) were housed separately in cages before experiments with the temperature of 23°C (22–24°C) and humidity of 40% (38%–42%) in a 12/12‐h light–dark environment with food and water ad libitum.

### 2.2 Stereotaxic surgery

After being anesthetized with 1.2% isoflurane and fixed on the stereotaxic apparatus, mice were exposed of skull and cleaned with 10% hydrogen peroxide. During the surgery, the mice were maintained warm by a heating plate. After viruses were microinjected (described later in this section) into the LHA (AP: −1.6 mm, ML: 0.4 mm, DV: −4.5 mm) using a Nanoject III injector (Drummond Scientific) at a rate of 23 nL/min, an optical fiber (diameter, 300 μm, Inper, Hangzhou, China) was implanted into the ipsilateral LHb (AP: −1.6 mm, ML: −0.4 mm, DV: −2.5 mm) for optogenetic experiments, or a guide cannula (RWD, Inc.) for chemogenetic or pharmacological manipulations. To obtain EEG recording, three stainless‐steel screws were anchored to the skull as electrodes: the positive and negative electrodes on the left and right sides of the head, respectively, or vice versa (AP: −1.5 mm, ML: −1.5 mm) and a reference electrode at the back of the head (AP: −5.5 mm, ML: 0 mm). After surgery, mice were housed for recovery for 7 days. A total of 3 weeks was required for the expression of virus in the brain.

### 2.3 Virus and drugs

Viruses rAAV2/9‐EF1a‐DIO‐mCherry, rAAV2/9‐EF1a‐DIO‐ChR2/NpHR‐mCherry, and rAAV2/9‐EF1a‐DIO‐hM3Dq/hM4Di‐mCherry were applied for the optogenetic and chemogenetic experiments. rAAV2/9‐EF1a‐DIO‐GCaMP6s was constructed for in vivo fiber photometry. For selective OxR2 knockdown, rAAV‐EF1a‐EGFP‐U6‐Loxp‐CMV‐mCherry‐loxp‐shRNA (scramble)/rAAV‐EF1a‐EGFP‐U6‐Loxp‐CMV‐mCherry‐loxp‐shRNA (OxR2‐shRNA) viruses were microinjected into the LHb. All viruses mentioned above were supplied by Brain VTA Technology Co., Ltd.

For pharmacological manipulations, OA and OB (Tocris Bioscience) dissolved in saline (333 pmol/μL) and the OxR1 antagonist SB334867 and OxR2 antagonist TCS‐OX2‐29 (Tocris Bioscience) dissolved in 5% dimethyl sulfoxide (Sigma‐Aldrich) (33.3 μg/μL) were injected into the LHb, separately. For chemogenetic regulation, clozapine N‐oxide (CNO, 5 μM/0.2 μL/side Cayman Chemical) or vehicle (saline) was administered by the guide cannula into the LHb 30 min before anesthesia. The microinjection speed was controlled using a micropump (Pump 11 Plus; Harvard Apparatus) at the rate of 0.1 μL/min.

### 2.4 Optogenetic stimulation

During deep anesthesia, mice remained unconscious and lost the righting reflex. The deep anesthesia was achieved by controlling the inhalational concentration of sevoflurane at around 2.4% under 100% O_2_ at a flow rate of 1.5 L/min, during which mice presented the steady burst‐suppression ratio (BSR) on the EEG up to 60–70%. Burst‐suppression pattern of EEG is a fundamental characteristic of a deeply inactivated brain during anesthesia. Under deep anesthesia, optogenetic manipulation was applied. The 2‐min blue laser (473 nm, 20 Hz, 30 ms, 15 mW from tips, Thinker Tech) was applied for activation, whereas 2‐min yellow laser (594 nm, 1 Hz, 1 s, 10 mW from tips, Thinker Tech) was applied for inhibition. Light anesthesia was achieved by administering the inhalational concentration of sevoflurane at 1.4–1.7% to avoid the presentation of burst suppression waves. During the state of light sevoflurane anesthesia, blue light (473 nm, 20 Hz, 30 ms, 15 mW from tips, for 120 s) was used to activate neurons. The position of each optical fiber tip was verified experimentally, and the EEG spectrogram was simultaneously recorded.

### 2.5 Fiber photometry for recording calcium signals

We attached the optical fiber to the fluorescence photometer (Thinker Tech) and confirmed the laser intensity at the fiber tip was between 30 and 40 μW persistently. The average ΔF/F values were calculated by a custom‐written MATLAB code, as (F duration‐F baseline)/F baseline, in which F baseline was the mean calcium signal before time zero.

### 2.6 Behavioral assessment

Mice were placed in a horizontal cylindrical observation cage (length, 45 cm; diameter, 12 cm) and anesthetized with 2.4% sevoflurane under 100% O_2_ at a flow rate of 1.5 L/min. During induction, the cylindrical chamber was rotated by 90° at each 15‐s interval to evaluate the righting reflex. When a mouse was unable to turn itself prone onto four paws within 15 s, this was considered to exhibit a loss of righting reflex (LORR), and the duration from the onset of anesthesia to LORR was recorded as the induction time. The animals were then continuously anesthetized for 30 min. As soon as the sevoflurane ceased, the righting reflex was checked by turning the cage by 90° every 15 s and the time from termination of sevoflurane to that of the righting reflex with all four paws touching the floor was recorded as the emergence time.

For a continuous sedative state of sevoflurane, each mouse was placed in the chamber and was allowed to inhale 2.4% sevoflurane for 20 min, after which the concentration of inhaled sevoflurane was decreased to 1.4%. If the mouse showed any signs of RORR, the sevoflurane concentration was increased in 0.1% increments until the mouse maintained LORR for 20 min successively at a still concentration. During this period, the concentration of sevoflurane was mildly varied (1.4–1.7%) depending on the behavioral variability of each mouse. Arousal responses to opto‐stimulation during the sedative state of sevoflurane were scored by technicians blinded to the experiments. According to the analysis of video recordings, technicians scored the spontaneous movements of the head, whiskers, and legs in intensity as absent (0), mild (1), or forceful (2). With regard to the righting and walking status of each animal during opto‐stimulation, retention of LORR was scored as 0, and the appearance of RORR was scored as 2. Subsequently, the RORR scoring was performed as follows: walking with no further movements, 0; crawling without the abdomen off the chamber bottom, 1; or walking with the abdomen off the chamber bottom, 2. The total score was the sum of the scores for body movements, righting, and walking.^17^

### 2.7 EEG recording and analysis

Electroencephalogram signals were continuously recorded by the PowerLab 16/35 amplifier system and LabChart Pro version 8.0.10 software (AD Instruments). Raw EEG data were digitized at a rate of 1000 Hz and were bandpass filtered at 0.3–50 Hz. The burst‐suppression ratio (BSR), total power percentage, and spectrum were analyzed using MATLAB (R2014a; MathWorks).

To calculate the BSR, the EEG voltage threshold was set based on the amplitude of suppression of each animal. If the amplitude of EEG was lower than the threshold for 0.5 s, it was defined as a suppression event and assigned a value of 1. Otherwise, signals above the threshold were defined as burst events and were assigned a value of 0. Finally, the BSR was calculated as the percentage of suppression events for 2 min before and during optical stimulation.^11^, ^18^

### 2.8 Immunohistochemistry

Immunofluorescence assay was performed as described previously.^19^, ^20^ In specific, brain slices were incubated with anti‐OxR2 (1:200, GeneTex) or orexin‐A antibody (1:200, MAB763, R&D Systems, USA) for 48 h, and then incubated with Alexa488 secondary antibody (1:1000, 711–545‐152, Jackson Immuno Research, USA) at room temperature for 2 h.

### 2.9 Electrophysiology

Mice were anesthetized with isoflurane and intracardially perfused with an ice‐cold cutting solution containing (in mM) 92 NMDG, 2.5 KCl, 1.25 NaH_2_PO_4_, 30 NaHCO_3_, 20 HEPES, 25 glucose, 2 thiourea, 5 Na‐ascorbate, 3 Na‐pyruvate, 0.5 CaCl_2_, and 10 MgSO_4_. Coronal brain slices containing LHA (300–350 μm thickness) were obtained in ice‐cold cutting solution bubbled with 95% O_2_ and 5% CO_2_ with a VT1200S Vibratome (Leica Microsystems). Slices were recovered in an interface chamber with artificial cerebrospinal fluid (ACSF) containing (in mM) 124 NaCl, 2.5 KCl, 1.25 NaH_2_PO_4_, 24 NaHCO_3_, 12.5 glucose, 5 HEPES, 2 CaCl_2_, and 2 MgSO_4_, bubbled with carbogen at 34°C for 45 min and kept at room temperature until recordings started. Slices were then transferred to recording chamber and continuously perfused with ACSF at the rate of 1‐3 mL per minute. Slices were visualized on an upright fixed‐stage microscope (Olympus) which is equipped with epifluorescence and infrared‐differential interference contrast (DIC) illumination to identify fluorescently tagged cells as well as optogenetic stimulation. Recording electrodes (3–6 MΩ resistance) were pulled from borosilicate glass and filled with potassium‐based intracellular solution containing (in mM) 145 mM K‐Gluconate, 10 mM HEPES, 1 mM EGTA, 2 mM Mg‐ATP, 0.3 mM Na_2_‐GTP, and 2 mM MgCl_2_.

Whole‐cell patch‐clamp recordings were performed and action potential (AP) were recorded in current‐clamp mode. To evoke APs from neurons expressing channelrhodopsin (ChR2) or inhibiting the firing activity from neurons expressing eNpHR3.0, 470 nm blue light (5‐50 Hz, 20 ms) and 593.5 nm yellow light (1 Hz, 1 s) were delivered via an optical fiber coupled to a LED light source above the recording cell. For chemogenetic experiment, positive or negative current (5–15 pA) was injected into the cell under current‐clamp mode to induce a steady firing activity, CNO (100 μM) was added in ACSF for bath application. Data were filtered and acquired using Axon 700B Amplifier (Molecular Devices) and pClamp 10 software (Molecular Devices, version 10.6).

### 2.10 Statistical analysis

All statistical analyses were performed with Prism 8.0.1 (GraphPad) and MATLAB (MathWorks). Data from all animals confirmed the viral expression were included in the statistical analysis. All data, except for arousal scores, are presented as mean ± standard error of mean (SEM). The normality of the data distributions was tested using the Shapiro–Wilk test. Statistical significance was assessed using the student's *t*‐test for comparison between two groups. Two‐way ANOVA followed by Bonferroni correction was used to evaluate statistical differences in the chemogenetic experiments. Behavioral scores for arousal are presented as median (25–75th percentile) and were analyzed using the Mann–Whitney *U*‐test.

## 3 RESULTS

### 3.1 Stimulation of the orexinergic terminals in the LHb promotes the emergence of mice from sevoflurane anesthesia

**FIGURE 1:**
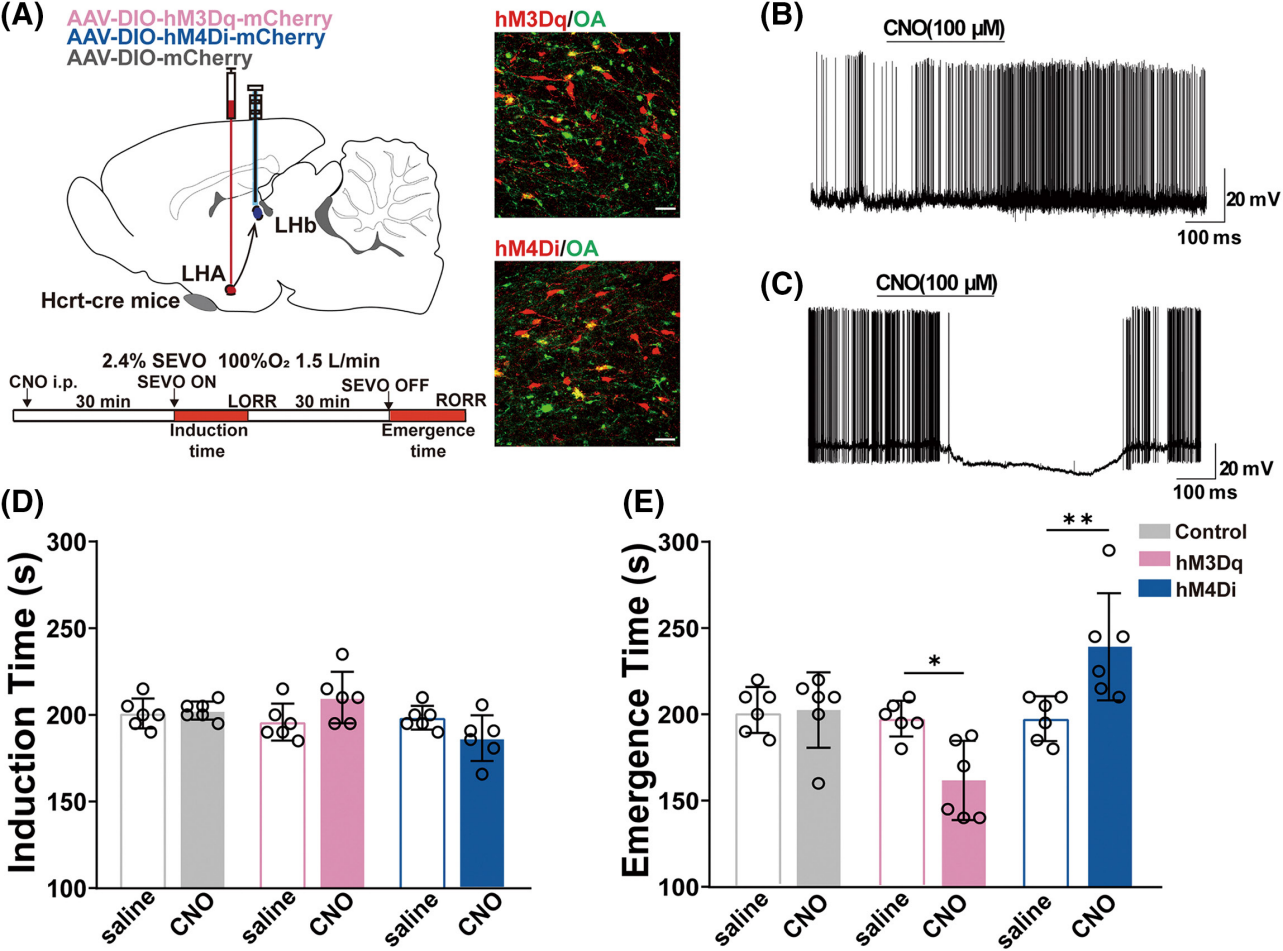
Chemogenetic modulation of orexinergic terminals in the LHb regulated the emergence from sevoflurane anesthesia. (A) Left, top, schematic illustration of virus injection and cannula location. Left, bottom, experimental protocol of assessment of time to LORR and RORR. Right, verification of virus expression (red: hM3Dq/hM4Di‐mCherry; green: OA). (B, C) Ex vivo electrophysiological confirmation of hM3Dq (B) or hM4Di (C) virus efficiency. (D, E) Induction time (D) and emergence time (E) of mice with chemogenetic activation or inhibition at the orexinergic terminals in the LHb. Activation of orexinergic terminals significantly shortened the emergence time (hM3Dq: 159.17 ± 8.1052 vs. 197.50 ± 4.2328, *p* = 0.0146); inhibition of orexinergic terminals significantly prolonged the emergence time (hM4Di: 239.17 ± 12.6765 vs. 197.50 ± 5.2836, *p* = 0.0061). Data are shown as mean ± SEM, *n* = 6 per group, *p < 0.05, **p < 0.01, two‐way ANOVA followed by Bonferroni correction. CNO, clozapine N‐oxide; LHA, lateral hypothalamic area; LHb, lateral habenula; LORR, loss of righting reflex; RORR, recovery of righting reflex; SEVO, sevoflurane.

To investigate the effect of orexinergic terminals in the LHb on the anesthetic effect of sevoflurane, we chemogenetically regulated orexinergic projections from the LHA to the LHb in the Hcrt‐cre mice. The successful viral expressions in LHb were morphologically verified after the experiments in all the animals included in the final analysis (Figure 1A). The efficiency of the virus in regulation of neuronal activity was verified by in vitro electrophysiology (Figure 1B,C). Vehicle or CNO was micro‐injected into the LHb 30 min before the initiation of 2.4% sevoflurane inhalation to activate or inhibit orexinergic terminals. Although chemogenetic stimulation of orexinergic terminals in the LHb showed little influence on the duration time of anesthesia induction (Figure 1D), activation of the orexinergic projections to the LHb shortened the emergence time compared with the control group (Figure 1E, *p* = 0.0146), while the inhibition of this projection prolonged the time of emergence (Figure 1E, *p* = 0.0061).

### 3.2 Activation of orexinergic terminals in the LHb reduces the burst‐suppression pattern of EEG during deep sevoflurane anesthesia

**FIGURE 2:**
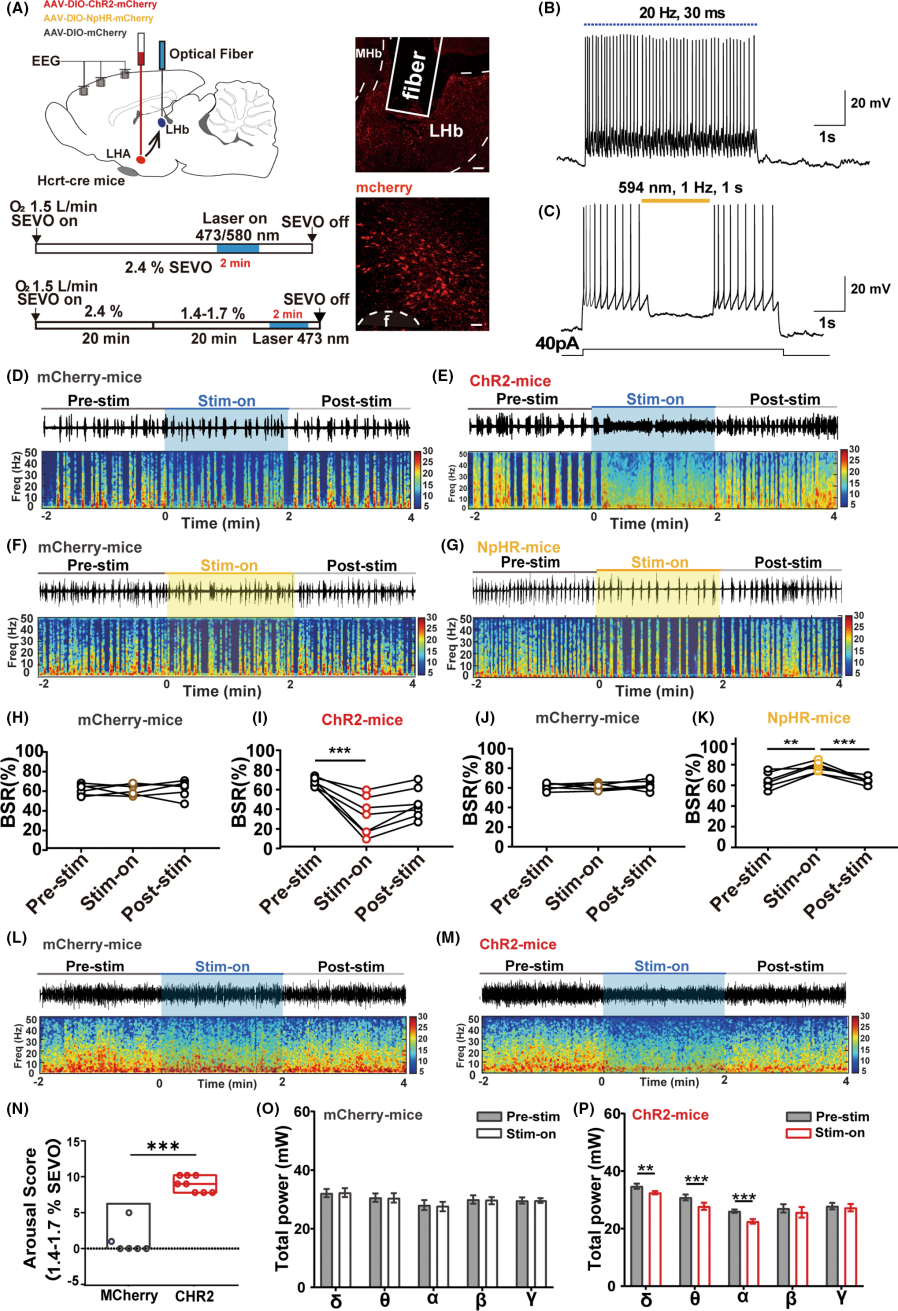
Optogenetic activation of orexinergic terminals in the LHb induced cortical activation from deep sevoflurane anesthesia. (A) Left, top, schematic illustration of virus injection and optical fiber. Left, bottom, experimental protocol for optogenetic manipulation during burst‐suppression pattern of EEG with deep sevoflurane anesthesia and protocol for optogenetic activation during continuous sedation of sevoflurane anesthesia. The blue line represents the time window of optical stimulation. Right, verification of virus expression (red: ChR2/NpHR‐mCherry) in the LHA and the location of optical fiber in the LHb. (B, C) Ex vivo electrophysiological confirmation of ChR2 (B) or NpHR (C) virus efficiency. (D–G) Representative EEG traces (above) and corresponding power spectra (below) before, during, and after optical stimulation under 2.4% sevoflurane anesthesia in the ChR2 group (E), NpHR group (G) and the mCherry (D, F) group. (H) Optical activation of mCherry group had no effect on the BSR (pre‐stim vs. stim‐on: 60.15 ± 3.117% vs. 59.73 ± 2.893%, *p* = 0.9242, *n* = 5). (I) Activation of orexinergic terminals in the LHb significantly reduced the BSR during anesthesia maintenance (pre‐stim vs. stim‐on: 67.48 ± 1.695% vs. 31.69 ± 7.514%, *p* = 0.0006, *n* = 7). (J) Optical inhibition of mCherry group had no effect on the BSR (pre‐stim vs. stim‐on: 61.41 ± 1.852% vs. 61.77 ± 1.522%, *p* = 0.9810, *n* = 6). (K) Inhibition of orexinergic terminals in the LHb significantly increased the BSR during anesthesia maintenance (pre‐stim vs. stim‐on: 64.78 ± 3.332% vs. 77.54 ± 1.974%, *p* = 0.0081, stim‐on vs. post‐stim: 77.54 ± 1.974% vs. 65.40 ± 1.420%, *p* = 0.0005, *n* = 6). (L, M) representative EEG traces (top) and corresponding power spectra (bottom) before, during, and after optical stimulation during continuous sedation of sevoflurane anesthesia in the ChR2 group (M) and the mCherry group (L). The blue shadow represents the time window of optical stimulation. (N) activation of the orexinergic terminals in the LHb induced immediate behavioral emergence in the ChR2 group (mCherry vs ChR2 group:1 ± 0.816% vs. 9 ± 0.378% *p* < 0.001). (O, P) comparison of the spectral power percentage for 1 min before and during optical activation in mCherry (O) group and ChR2 group (P). Data are shown as median (25–75th percentile) for arousal scores, and as mean ± SEM for the other data, *n* = 5–8 per group, **p < 0.01, and ***p < 0.001.

To further investigate the effect of the LHb orexinergic terminals on deep anesthesia maintenance, we modulated its excitability using an optogenetic approach when animals anesthetized with 2.4% sevoflurane were under deep maintenance state and exhibited constant burst suppression patterns of the EEG, an indicator for deep anesthesia (Figure 2A). For this purpose, the excitatory ChR2‐containing virus or inhibitory NpHR‐containing virus was specifically expressed in the LHA orexinergic neurons, and the optical fiber was secured above the LHb area. The position of each optical fiber tip was verified after experiments (Figure 2A). The effectiveness of optical stimulation was confirmed successful in the acute slices by the electrophysiological tests (Figure 2B,C).

When the burst suppression pattern was regularly induced for at least 10 min, the optical activation of the orexinergic terminals in the LHb significantly decreased BSR from 67.48% to 31.69% (*p* = 0.0006). As the optical stimulation ceased, the BSR returned to the pre‐stimulation state (Figure 2E,I). In comparison, the BSR was increased from 64.78% to 77.54% (*p* = 0.0081) by the photo‐inhibition of the LHb orexinergic projections, and also returned to the pre‐stimulation state once the light was turned off (Figure 2G,K). As shown in Figure 2D,F,H,J, the burst suppression pattern of the mCherry group did not change across the optical stimulation (*p* = 0.9242). Therefore, the orexinergic terminals in the LHb might be involved in the regulation of the sevoflurane anesthetic depth.

### 3.3 Activation of the orexinergic terminals in the LHb induces cortical activation and wakeful manifestation of mice from light anesthesia

We further investigated the regulatory effect of the LHb orexinergic terminals during light anesthesia (steady sedation with 1.4–1.7% sevoflurane) using acute optical stimulation (Figure 2). The optical activation of the orexinergic terminals in the LHb induced an immediate behavioral wakeful manifestation in ChR2‐expressing mice, including the appearance of movements of legs, heads, and whiskers, as well as righting and walking (Table S1). In contrast, mice in the controlled mCherry group exhibited none of the above behavioral manifestations during photostimulation. The overall arousal score of the behaviors was much higher in the ChR2 group than the mCherry group (*p* < 0.001).

In addition, photostimulation of the orexinergic terminal in the LHb also induced a brain‐state transition in EEG from a slow‐wave activity pattern to a fast‐wave activity pattern in ChR2 mice (Figure 2M), but not in mCherry mice (Figure 2L). Spectral analysis of the EEG revealed that acute photostimulation induced a significant decrease in delta power (Figure 2P, *p* = 0.0061, *n* = 6), theta power (Figure 2P, *p* = 0.0002, *n* = 6), and alpha power (Figure 2P, *p <* 0.0001, *n* = 6). After the cessation of laser stimulation, the EEG spectral signatures gradually returned. Collectively, these findings indicated that activation of the LHb orexinergic terminals was sufficient to induce cortical activation and behavioral emergence in mice from the light anesthesia state.

### 3.4 LHb orexinergic terminals exert the emergence‐promotive behavioral effect in sevoflurane anesthesia through the OA/OB and orexin‐2 receptor

**FIGURE 3:**
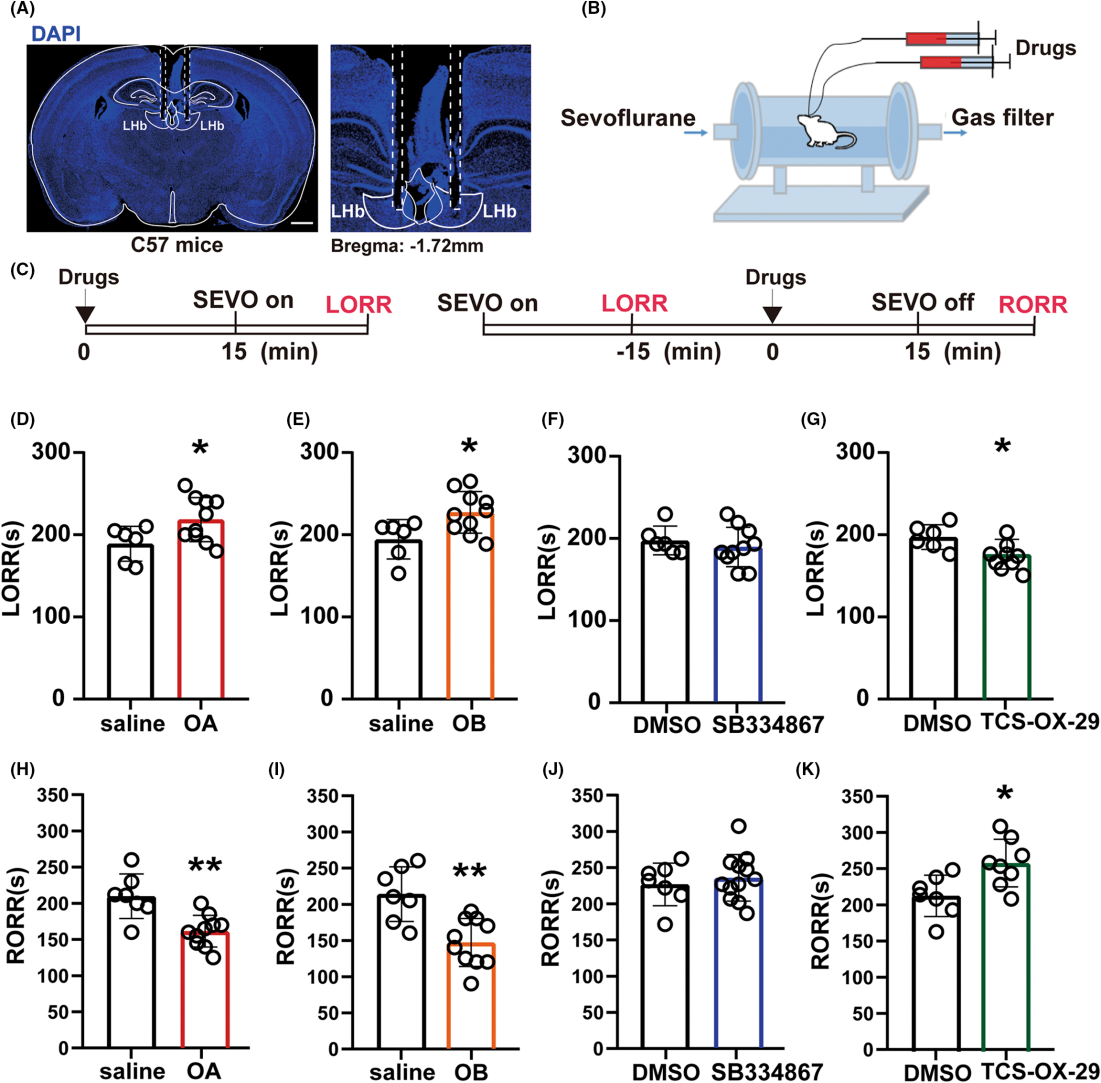
Orexin‐A/B and orexin‐2 receptor in the LHb participated in induction and emergence of sevoflurane anesthesia procedure. (A) The microinjection sites in the bilateral LHb. (B) The diagram of the LHb microinjection and the anesthesia behavior observation box. (C) The pharmacological experiment protocols for determining the LORR and RORR. (D, H) Microinjection of orexin‐A into the LHb prolonged induction time (D, 218.50 ± 11.016% vs. 189.17 ± 8.533%, *p* = 0.0401) and shortened emergence time (H, 161.50 ± 6.914% vs. 209.71 ± 11.680%, *p* = 0.0018). (E, I) Microinjection of orexin‐B into the LHb prolonged induction time (E, 223 ± 7.860% vs. 190.83 ± 93.610%, *p* = 0.023) and shortened emergence time (I, 147 ± 10.467% vs. 213.86 ± 14.255%, *p* = 0.0015). (F, J) Microinjection of orexin‐1 receptor antagonist SB334867 into the LHb had no effect on induction time (F, 181.5 ± 7.343% vs. 189.17 ± 6.882%, *p* = 0.4946) or emergence time (J, 233.917 ± 9.250% vs. 225 ± 11.073%, *p* = 0.5546). (G, K) Microinjection of orexin‐2 receptor antagonist TCS‐OX2‐29 into the LHb shortened the induction time (G, 172.78 ± 7.222% vs. 202.50 ± 8.441%, *p* = 0.0201) and prolonged the emergence time(K, 249.167 ± 9.610% vs. 221.00 ± 13.095%, *p* = 0.0225). Data are shown as the mean ± SEM, *n* = 6–10 per group, *p < 0.05, **p < 0.01. DMSO, dimethyl sulfoxide; LHb, lateral habenula; OA, orexin‐A; OB, orexin‐B; SEVO, sevoflurane.

To explore the subtypes of orexin peptides and their receptors in the LHb involved in modulation of sevoflurane anesthesia, we performed pharmacological manipulations to the bilateral LHb under sevoflurane anesthesia at different time points (Figure 3C). Microinjection of OA and OB showed a longer induction time as compared with the control group (OA vs. Con: 218.50 ± 11.016% vs. 189.17 ± 8.533%, *p* = 0.0401; OB vs. Con: 223.00 ± 7.860% vs. 190.83 ± 93.610%, *p* = 0.023; Figure 3D,E) when the injections were performed 15 min before sevoflurane inhalation, and shorter emergence time than the control group when the injections were performed 15 min before the sevoflurane cessation (OA vs. Con: 161.50 ± 6.914% vs. 209.71 ± 11.680%, *p* = 0.0018; OB vs. Con: 147 ± 10.467% vs. 213.86 ± 14.255%, *p* = 0.0015; Figure 3H,I).

As shown in Figure 3F,J, there was no significant difference between the selective OxR1 antagonist SB‐334867A (SB) group and the control group in both induction time (SB vs. Con: 181.5 ± 7.343% vs. 189.17 ± 6.882%, *p* = 0.4946) and emergence time (SB vs. Con: 233.917 ± 9.250% vs. 225 ± 11.073%, *p* = 0.5546). However, the selective OxR2 antagonist TCS‐OX2‐29 (TCS) group showed a shorter induction time (TCS vs. Con: 172.78 ± 7.222% vs. 202.50 ± 8.441%, *p* = 0.0201; Figure 3G) and longer emergence time (TCS vs. Con: 249.167 ± 9.610% vs. 221.00 ± 13.095%, *p* = 0.0225; Figure 3K) than the control group.

### 3.5 Administration either orexin A (OA) or orexin B (OB) at LHb decreases the depth of sevoflurane anesthesia as detected by EEG

**FIGURE 4:**
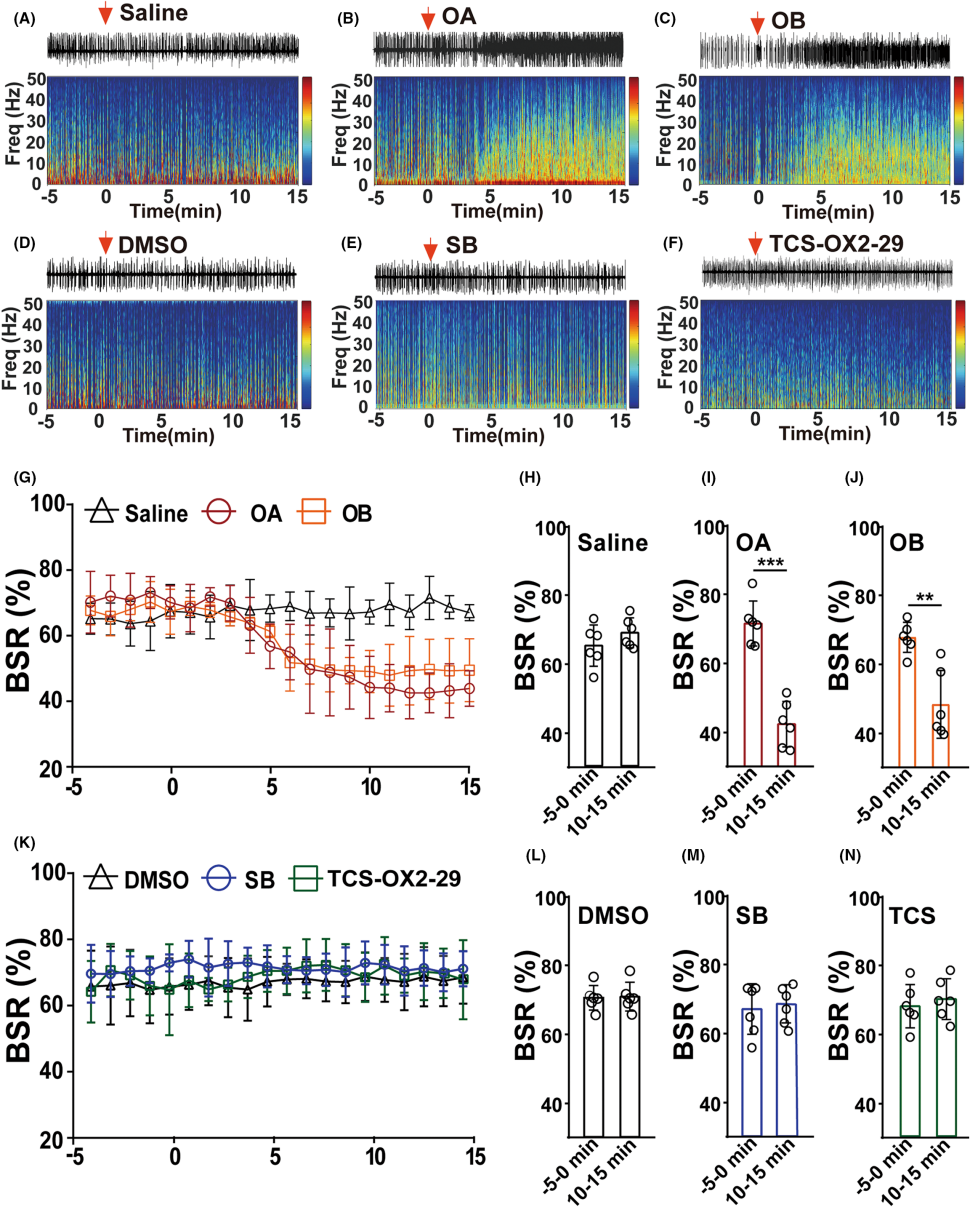
Exogenous orexins in the LHb reduced the burst‐suppression pattern of EEG during the deep sevoflurane anesthesia. (A–F) Representative raw EEG traces (Top) and EEG spectra (Bottom) after microinjection of saline (A), orexin‐A (B), orexin‐B (C), DMSO (D), orexin‐1 receptor antagonist SB334867 (E), or orexin‐2 receptor antagonist TCS‐OX2‐29 (F) into the LHb. (H–J) Microinjection of orexin‐A or orexin‐B into the LHb decreased the BSR during 2.4% sevoflurane anesthesia maintenance. (I, OA: −5–0 min vs. 10–15 min: 71.14 ± 2.657% vs. 42.02 ± 2.685%, *p* < 0.0001; J, OB: −5–0 min vs. 10–15 min: 67.57 ± 1.777% vs. 48.17 ± 4.004%, *p* = 0.0013). (L–N) Microinjection of SB or TCS‐OX2‐29 into the LHb had no effect on BSR during 2.4% sevoflurane anesthesia maintenance. (M, SB: −5‐0 min vs. 10–15 min: 65.63 ± 2.890% vs. 67.10 ± 2.187%, *p* = 0.693; N, TCS‐OX2‐29: −5–0 min vs. 10–15 min: 66.43 ± 2.478% vs. 68.42 ± 2.334%, *p* = 0.571). (G, K) average BSR per minute 5 min before and 15 min after microinjection of orexin (G) and orexin receptor antagonists (K) into the LHb. Data are shown as mean ± SEM, *n* = 6 per group, **p < 0.01, ***p < 0.001. DMSO, dimethyl sulfoxide; OA, orexin‐A; OB, orexin‐B; SB, SB334867; TCS, TCS‐OX2‐29.

We further microinjected OA, OB, SB334867, and TCS‐OX2‐29, respectively, into the LHb to observe their individual regulatory effects during maintenance of deep anesthesia (Figure 4A–F). Interestingly, both OA and OB administration reduced the number of burst‐suppression waves, but neither SB334867 nor TCS‐OX2‐29 microinjection changed the EEG spectrogram, as shown by BSR data (Figure 4G–N). Notably, the decreases in BSR induced by OA (−5–0 min vs. 10–15 min: 71.14 ± 2.657% vs. 42.02 ± 2.685%, *p* < 0.0001, Figure 4I) and OB (−5–0 min vs. 10–15 min: 67.57 ± 1.777% vs. 48.17 ± 4.004%, *p* = 0.0013, Figure 4J) lasted for at least 15 mins (Figure 4G), which was much longer than the effect of the optogenetic activation of LHb orexinergic terminals (Figure 2).

### 3.6 Knockdown of OxR2 in GAD2 neurons but not in glutamate neurons of the LHb prolongs emergence from sevoflurane anesthesia

**FIGURE 5:**
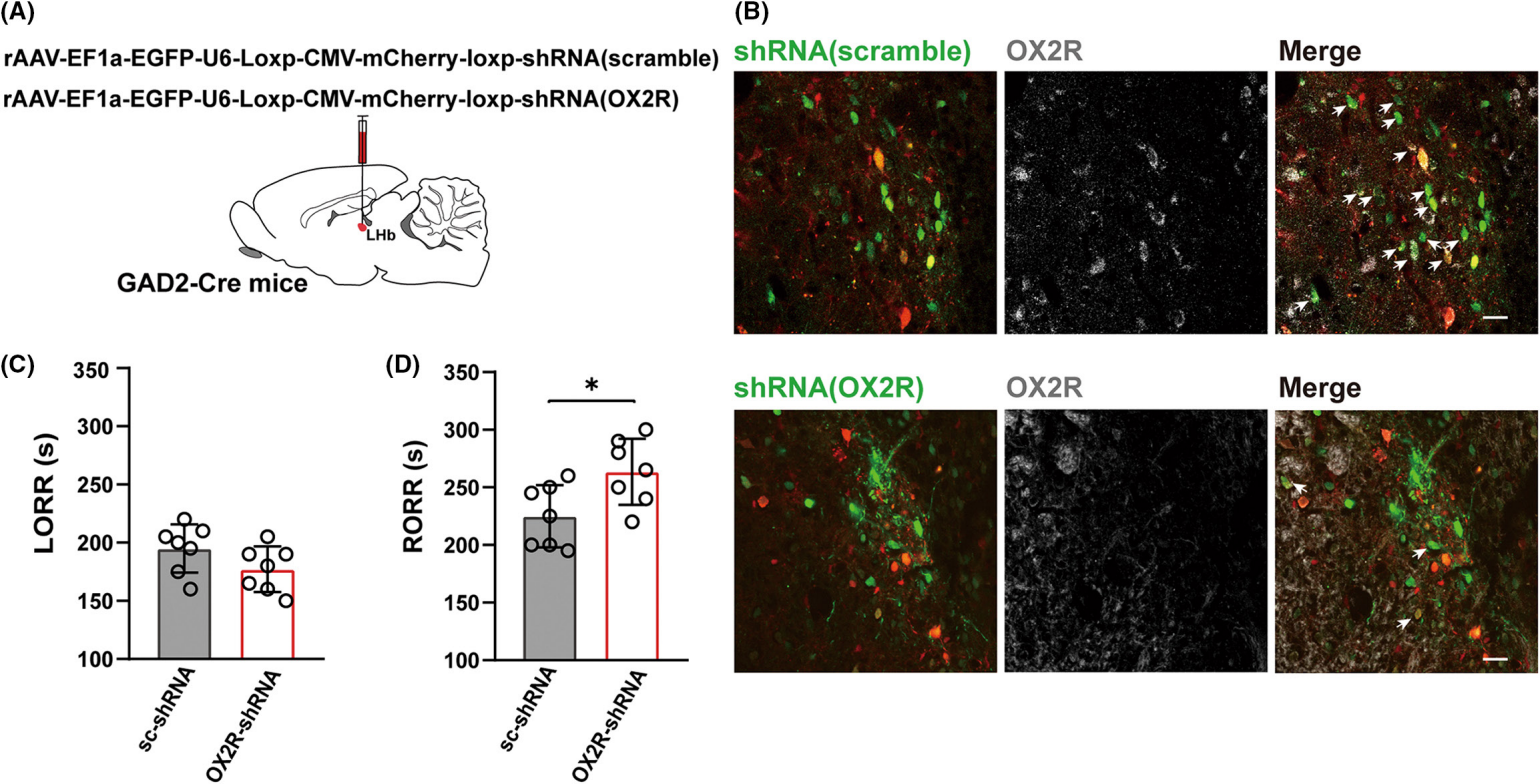
Specific knockdown of OX2R at the GAD2 neurons in the LHb prolonged emergence from sevoflurane anesthesia. (A) The representative expression images of shRNA virus in the LHb. Scramble shRNA virus (top left, green) or OX2R shRNA virus (bottom left, green), OX2R expression (top/bottom middle, gray) and the overlapping neurons (top/bottom right) are shown in the LHb of the GAD2‐Cre mice. (B) Specific knockdown of OX2R at GAD2 neurons in the LHb had no effect on the induction time (195.00 ± 7.868% vs. 177.143 ± 7.389%, *p* = 0.1239). (C) Specific knockdown of OX2R of GAD2 neurons in the LHb prolonged emergence from sevoflurane anesthesia (225.00 ± 10.235% vs. 263.571 ± 10.841%, *p* = 0.0238). Data are shown as mean ± SEM, *n* = 7 per group, *p < 0.05.

Previous studies confirmed that OxR2 in the LHb is highly expressed in GAD2‐positive GABAergic neurons. Therefore, we selectively knocked down OxR2 in LHb GABAergic neurons using the microRNA interference technique in a GAD2‐cre mouse line. As shown in Figure 5, OxR2 expression in the GAD2 neurons of the LHb was significantly decreased. Statistically, the colocalization of the OxR2 and GAD2 decreased from 69.35 ± 4.926% to 20.33 ± 3.662% in the OxR2‐shRNA group compared with the scramble group (Figure S1, *p <* 0.001). OxR2 knockdown in LHb GAD2‐expressing neurons did not alter the average induction time (*p* = 0.1239) but markedly extended the time to RORR (*p* = 0.0238).

To clarify the effect of OxR2 in LHb glutamatergic neurons on anesthesia regulation, we knocked down the expression of OxR2 in LHb glutamatergic neurons using the same strategy. Downregulation of OxR2 in LHb vesicular glutamate transporters 2 (vGlut2) neurons did not affect either the induction or the emergence procedures (Figure S2).

The above results indicate that projection of orexinergic neurons to LHb promoted arousal from sevoflurane anesthesia, potentially through their innervation to LHb GABAergic neurons via OxR2.

### 3.7 Activity of LHb GABA neurons is inhibited during sevoflurane anesthesia and recovered during emergence

**FIGURE 6:**
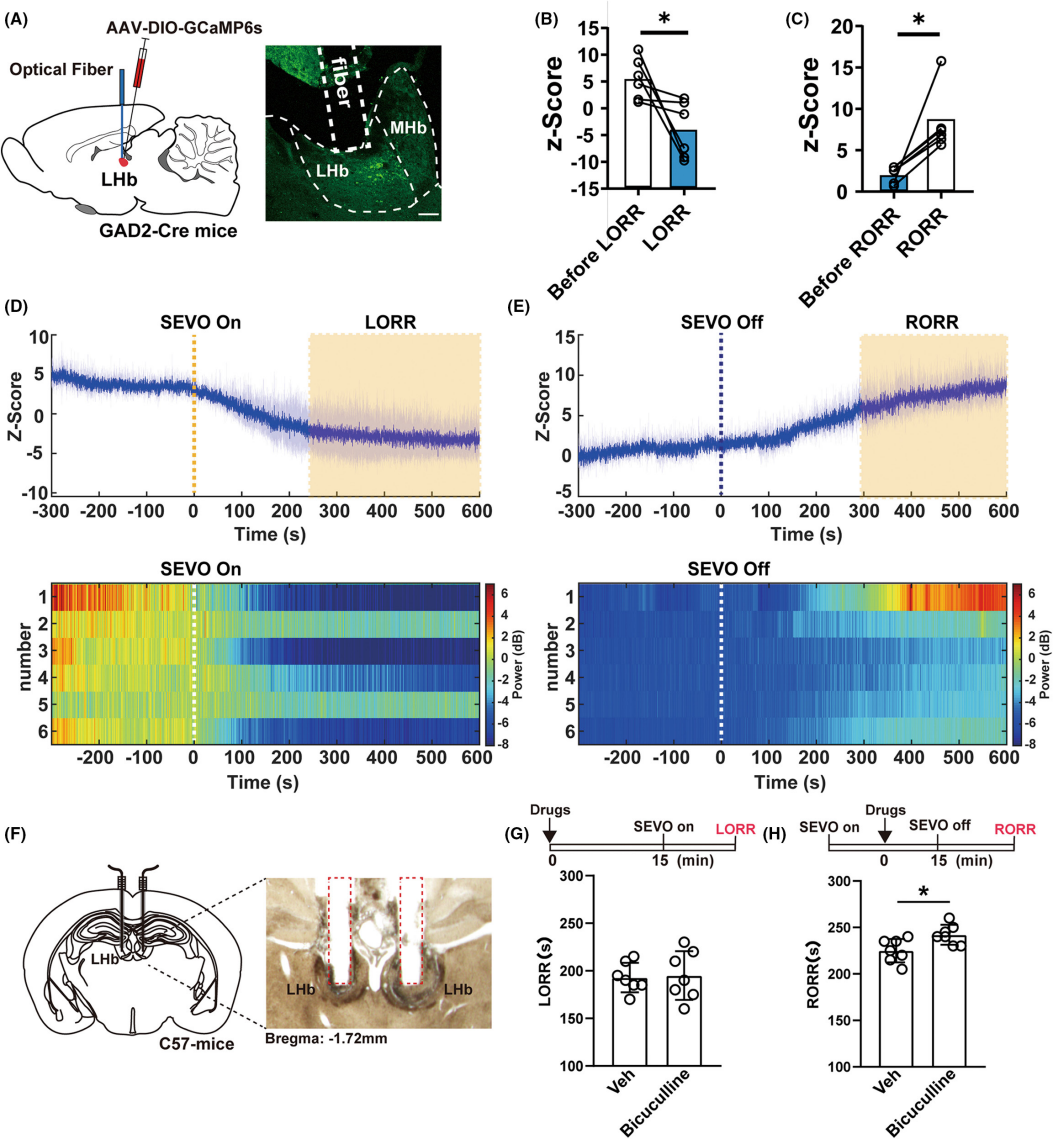
Sevoflurane anesthesia decreased the activities of GAD2 neurons in the LHb. (A) Left, schematic illustration of virus injection and position of optical fiber. Right, verification of virus expression and track of optical fiber. (B) LHb GAD2 neuronal activity was significantly reduced during loss‐of‐consciousness state (5.50 ± 1.575 vs. −4.09 ± 2.161, *p* = 0.0421). (C) Calcium signals of LHb GAD2 neurons were increased after the recovery of consciousness from sevofluane anesthesia (2.10 ± 0.406 vs. 8.28 ± 1.528, *p* = 0.016). (D) Top, the averaged calcium signal gradually decreased during the sevoflurane anesthesia induction (orange shaded area displays the unconscious state); bottom, the heatmaps of calcium signals during the sevoflurane anesthesia induction. (E) Top, the averaged calcium signal gradually increased during emergence from the sevoflurane anesthesia (orange shaded area displays the time duration of unconscious state); bottom, the heatmaps of calcium signals during the sevoflurane anesthesia emergence. (F) Microinjection sites of the GABA A receptor antagonist administration in the LHb. (G) Microinjection of GABA A receptor antagonist into the LHb had no effect on the induction time (195.00 ± 10.775% vs. 192.8 ± 5.861%, *p* = 0.8532). (H) Microinjection of GABA A receptor antagonist into the LHb prolonged emergence time (242.14 ± 4.062% vs. 225.00 ± 4.756%, *p* = 0.0179). Data are shown as mean ± SEM, *n* = 7 per group, *p < 0.05.

To clarify the changes in excitability of LHb GAD2‐expressing neurons during sevoflurane anesthesia, we injected AAV‐DIO‐GCaMP6s virus into the LHb of GAD2‐Cre mice (Figure 6A). The calcium signals of LHb GAD2‐expressing neurons gradually decreased after the initiation of sevoflurane anesthesia (Figure 6B,D) and then regained slowly after the cessation of anesthetic inhalation (Figure 6C,E), indicating that the dynamic changes in the excitability of GAD2‐expressing neurons were consistent with those in orexinergic neurons under sevoflurane anesthesia. As LHb GABAergic neurons have an inhibitory microcircuit to LHb glutamatergic neurons that have been reported to facilitate the general anesthesia effect, we wondered whether activation of LHb GABAergic neurons mediated anesthesia emergence via their local inhibitory effect. Therefore, we pharmacologically blocked the GABA A receptor at the LHb (Figure 6F) by microinjecting bicuculline (1 nmol in 100 nL). We found no change in induction time (bicuculline vs. Con:195.00 ± 10.775% vs. 192.86 ± 5.861%, *p* = 0.8532, Figure 6G), but there was a significant delay in emergence time (bicuculline vs. Con: 242.14 ± 4.062% vs. 225.00 ± 4.756%, *p* = 0.0179, Figure 6H).

## 4 DISCUSSION

In this study, we found that chemogenetic activation of orexinergic^LHA‐LHb^ projections could significantly facilitate emergence from sevoflurane anesthesia. Optogenetic activation of orexinergic terminals in the LHb not only induced cortical arousal in terms of the change in EEG signatures during deep anesthesia but also led to behavioral emergence during light anesthesia. We found that administration of two peptides of orexins, OA, and OB in LHb decreased the depth of anesthesia and promoted emergence from sevoflurane anesthesia mainly through OxR2 in GAD2‐expressing neurons but not glutamatergic neurons in the LHb. Therefore, we proposed that LHb GAD2‐expressing neurons mediated the emergence‐facilitative effect possibly through local inhibitory circuit on glutamatergic neurons.

LHb is one of the major projected targets of the orexin system. The orexin^LHA‐LHb^ pathway has been widely reported to be involved in motivated behavioral regulations.^7^, ^9^ Particularly, orexin terminals in the LHb induced aggressive behavior in male mice by activating LHb GAD2‐expressing GABA neurons, and this was mediated by the feedforward inhibition of LHb glutamatergic neurons.^14^ Previously we also reported that orexin neurons and their terminal in the LHb significantly alleviated depression‐like behaviors caused by chronic social defeat stress via activating the LHb glutamate neurons.^20^ In the current study, we firstly reported a substantial arousal‐facilitating effect of orexinergic terminals in the LHb during sevoflurane anesthesia by using chemogenetic, opotogenetic, and pharmacological approaches. Relevantly, Gelegen et al. found that blocking the LHb output caused natural sleep to be more fragmented, and this was largely reversed by systemic administration of dual orexinergic antagonist.^15^ Moreover, we previously found the stimulation of glutamatergic terminals in the LHb derived from the LHA, where orexin neurons are exclusively located, also induced similar facilitation of emergence during isoflurane anesthesia.^16^ It is worth noting that more than 50% of orexinergic neurons co‐release glutamate from their terminals,^21^, ^22^, ^23^ providing further support for the arousal effect of the orexin^LHA–LHb^ pathway during general anesthesia.

As previously reported, OxR1 is specifically activated by the OA, but OxR2 combines evenly with both OA and OB. Both OA and OB, or OxR1 and OxR2, have been reported to involve in anesthesia emergence.^11^, ^24^ In this study, administration of both OA and OB to the LHb prolonged the induction time of sevoflurane anesthesia, decreased the depth of anesthesia maintenance, and accelerated the arousal time, however, only OxR2 in the LHb was involved in the arousal‐promoting effect of orexinergic projections, indicating the selective innervation of the orexin system in the LHb during general anesthesia.

OxR1 and OxR2 are highly distributed in the LHb, and both glutamate and GABA neurons express these receptors. Although the number of glutamate neurons accounts for the vast majority in the LHb, OxR2 is almost exclusively expressed in LHb GAD2‐expressing GABA neurons, whereas fewer than 10% of LHb vGlut2‐expressing glutamate neurons are OxR2‐positive.^14^ Using microRNA interference techniques, we found that knocking down the expression of OxR2 on GAD2‐expressing GABA neurons delayed the arousal time of sevoflurane anesthesia, while OxR2 on vGlut2‐expressing glutamate neurons had no effect on anesthesia‐related behavior, indicating that OxR2 on vGlut2 neurons may not be involved in the regulation of sevoflurane anesthesia. It is interesting that the inhibitory GABA neurons could mediate an arousal effect, but this result coincide with previous studies that the excitatory glutamatergic neurons in the LHb mediated the anesthetic effects of propofol and isoflurane.^15^ The anesthesia‐facilitating effect of LHb glutamate neurons was speculated through their downstream of the rostromedial tegmental nucleus.^16^ As a matter of fact, Flanigan reported a feedforward local inhibitory regulation between the GAD2‐expressing GABA neurons and the vGlut2‐expressing glutamate neurons within the LHb, suggesting that GABA neurons activation‐induced glutamate neurons inhibition during anesthesia emergence may be involved in the pro‐arousal effect of orexinergic terminals in the LHb.^14^ To test this hypothesis, we recorded calcium signals of LHb GABA neurons over the whole sevoflurane anesthesia procedure and found the decrease of GABAergic activity during anesthesia and the recovery during emergence, which was consistent with the changes of excitability of orexin neurons and contrary to that of glutamate neurons in the LHb as reported previously.^16^ We also blocked the local GABAergic effect by administering the inhibitor of GABA A receptor and found the emergence from sevoflurane was prolonged. In consideration of the great number of glutamate neurons in the LHb, the arousal‐promoting effect of the LHb GABA neurons is rather likely to be mediated by the inhibition of LHb Glutamate neurons. Further investigations are needed to obtain more solid evidence.

The present study had some limitations. By administering exogenous OA and OB in the LHb, we confirmed the involvement of orexin peptides in regulating sevoflurane anesthesia, but the actual release of orexins in the LHb terminals still needs experimental confirmation. Orexin sensors may help to quantify the orexin release. Moreover, in addition to the local inhibitory projections, LHB GABA neurons also have long‐range projections in the brain; we did not fully clarify the downstream circuits of LHb GABA neurons in promoting arousal, which requires more studies in the future.

In conclusion, the present study provides evidence for the role of orexin^LH–LHb^ in facilitating emergence from sevoflurane anesthesia, and this effect was mediated by OxR2 in GAD2‐expressing GABA neurons. This study further expands on the effects of the LHb on general anesthesia. And, since LHb is highly associated with negative emotional behaviors, our findings could have implication regarding perioperative psychiatric disorders.

## AUTHOR CONTRIBUTIONS

Qianzi Yang and Hailong Dong initiated and supervised the study; Fang Zhou, Dan Wang and Tingting Tong performed the experiments; Xinxin Zhang, Ao Li and Sa Wang analyzed the EEG data; Huiming Li recorded and analyzed the electrophysiological data; Fang Zhou wrote the manuscript draft. Qianzi Yang, Haixing Zhong, and Hailong Dong revised the manuscript. All authors contributed to and approved the publication of this manuscript.

## FUNDING INFORMATION

National Natural Science Foundation of China, Grant Number: 82071554 to Qianzi Yang; Grant Number: 82030038 to Hailong Dong; Grant Number: 82101343 to Dan Wang.

## CONFLICT OF INTEREST STATEMENT

The authors state that there are no conflicts of interest to disclose.

## Supporting information


Figure S1.

Figure S2.

Table S1.

## Data Availability

The data that support the findings of this study are available from the corresponding author upon reasonable request.
