# Erratum

**DOI:** 10.1111/cns.14273

**Published:** 2023-05-29

Correction to “Orexinergic innervations at GABAergic neurons of the lateral habenula mediates the anesthetic potency of sevoflurane”

Zhou, F, Wang, D, Li, H, et al. Orexinergic innervations at GABAergic neurons of the lateral habenula mediates the anesthetic potency of sevoflurane. CNS Neurosci Ther. 2023; 29: 1332–1344. doi:10.1111/cns.14106

The authors would like to correct the title of this article to “*Orexinergic innervations at GABAergic neurons of the lateral habenula facilitates the emergence from sevoflurane anesthesia*”.

We apologize for this error.

