# Supplementary figures and images for: Orexinergic innervations at GABAergic neurons of the lateral habenula mediates the anesthetic potency of sevoflurane
Source: CNS Neurosci Ther. 2023 Feb 5;29(5):1332–44. doi: 10.1111/cns.14106 (PMC10068468; doi:10.1111/cns.14106)

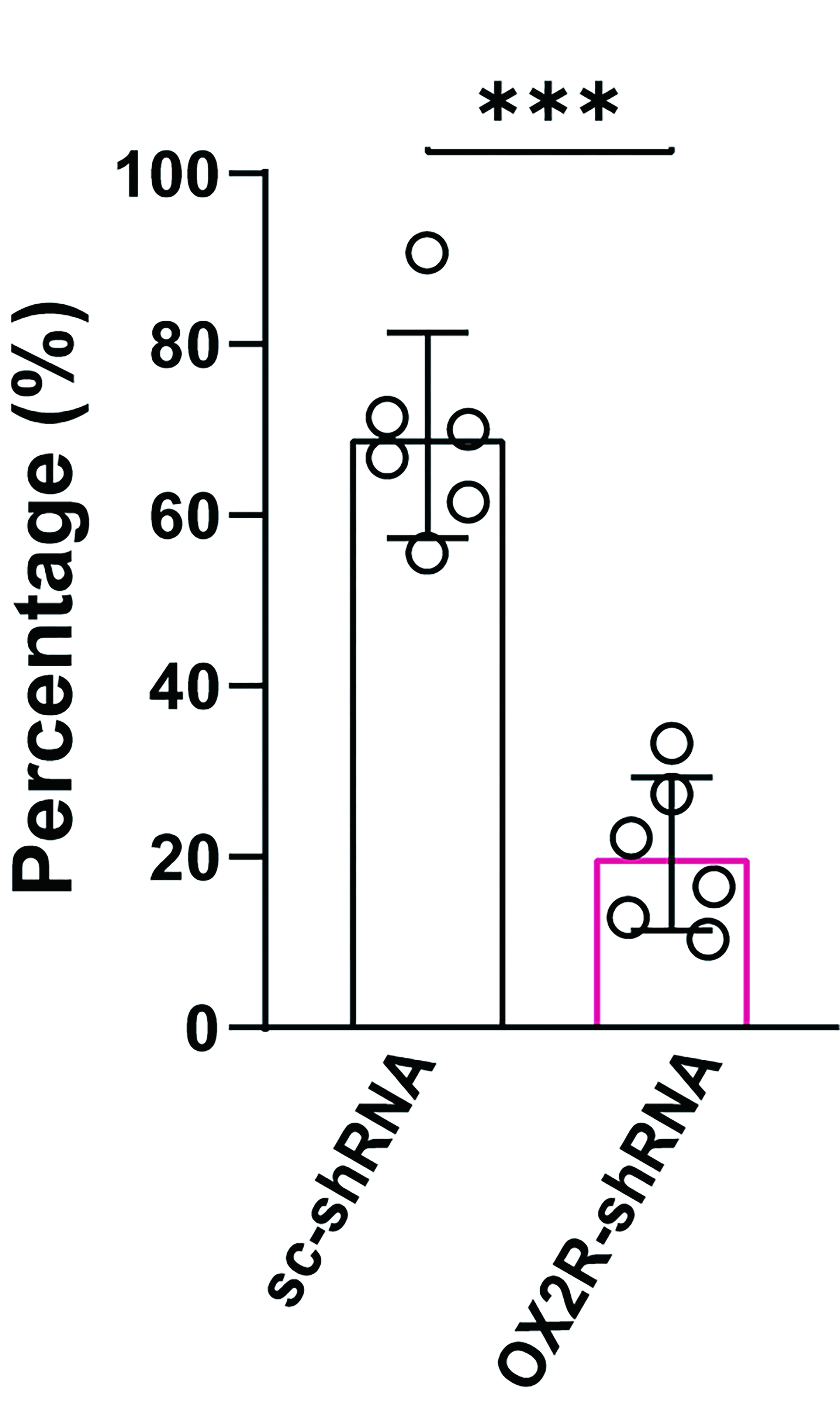

Supplement: Supplementary file 1 — Figure S1. [file CNS-29-1332-s003.tif]

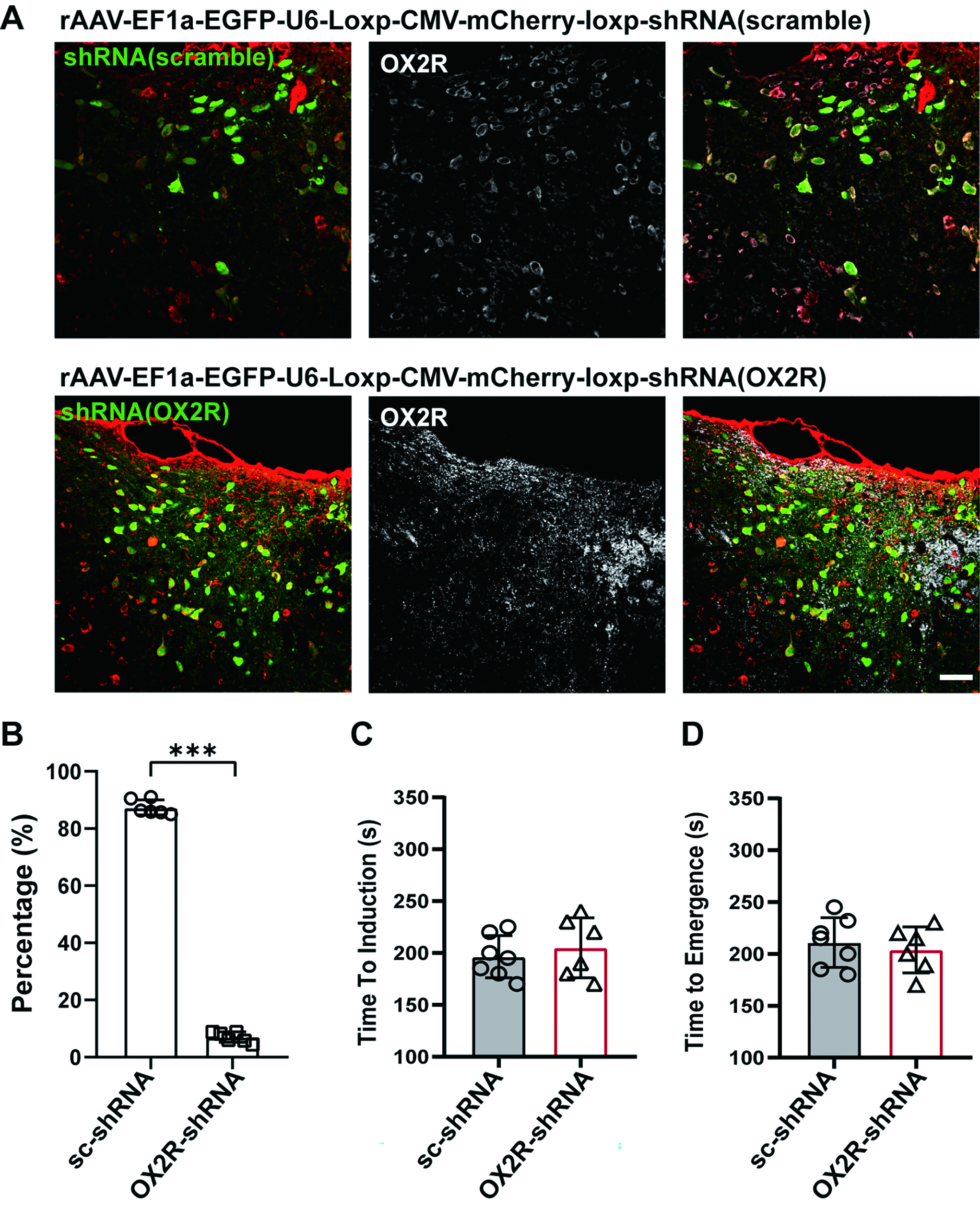

Supplement: Supplementary file 2 — Figure S2. [file CNS-29-1332-s001.tif]
