# Supplementary material for: Orexinergic innervations at GABAergic neurons of the lateral habenula mediates the anesthetic potency of sevoflurane
Source: CNS Neurosci Ther. 2023 Feb 5;29(5):1332–44. doi: 10.1111/cns.14106 (PMC10068468; doi:10.1111/cns.14106)
Supplement: Supplementary file 3 — Table S1. [file CNS-29-1332-s002.docx]

| **GROUP** | **Leg movement** | **Head movement** | **Whisker movement** | **Righting** | **Walking** | **Total score** |
| --- | --- | --- | --- | --- | --- | --- |
| ChR2-no1 | 2 | 2 | 2 | 2 | 1 | 9 |
| ChR2-no2 | 2 | 1 | 2 | 2 | 1 | 8 |
| ChR2-no3 | 2 | 2 | 2 | 2 | 2 | 10 |
| ChR2-no4 | 2 | 1 | 2 | 2 | 1 | 8 |
| ChR2-no5 | 2 | 2 | 2 | 2 | 2 | 10 |
| CHR2-no6 | 2 | 2 | 2 | 2 | 1 | 9 |
| ChR2-no7 | 2 | 1 | 2 | 2 | 1 | 8 |
| ChR2-no8 | 2 | 2 | 2 | 2 | 2 | 10 |
| Mcherry-no1 | 1 | 1 | 1 | 2 | 0 | 5 |
| Mcherry-no2 | 1 | 0 | 0 | 0 | 0 | 1 |
| Mcherry-no3 | 0 | 0 | 0 | 0 | 0 | 0 |
| Mcherry-no4 | 0 | 0 | 0 | 0 | 0 | 0 |
| Mcherry-no5 | 0 | 0 | 0 | 0 | 0 | 0 |
| Mcherry-no6 | 0 | 0 | 0 | 0 | 0 | 0 |

**SUPPLEMENTARY TABLE 1: Arousal behavior scores after the optogenetic activation of orexinergic terminals in the LHb during continuous sedation of sevoflurane anesthesia.**

Leg, head, and whisker movements, and the righting and walking status were scored for each animal.
